# An exploration of the elements of effective cultural healing in North America

**DOI:** 10.1192/j.eurpsy.2021.1819

**Published:** 2021-08-13

**Authors:** L. Mehl-Madrona, B. Mainguy

**Affiliations:** Medical Arts And Humanities Program, University of Maine, Orono, United States of America

**Keywords:** radical empathy, cultural healing, indigenous North Americans, spiritual transformation

## Abstract

**Introduction:**

How traditional cultural healing works is difficult for biomedical science to understand. Outcomes do occur that defy the conventional logic of materialistic, reductionistic cause-and-effect.

**Objectives:**

We aimed to understand how participants understood what happens in traditional cultural healing.

**Methods:**

We identified 26 cases of results in which improvement occurred beyond what biomedicine would expect from a placebo response. We interviewed the healers and their clients to understand their experience and how they saw what had happened.

**Results:**

Seven cases involved resolution of cancer; 2 cases, musculoskeletal disorders; 9 cases of rheumatological disorders; 8, other disorders. Each person spoke about the importance of spiritual transformation and described such an experience. They spoke about an attitude of the cultural healer that involved what could best be translated as radical empathy coupled with non-judgmental listening without interpretation. Many of healers had been initiated into their healing roles via a life-threatening illness that resolved when an extra-ordinary being(s) (a spirit or god, or God) entered their life world and became an integral part of their being. This was also a common description given by the participants for what had happened. The healers often described themselves as a hollow bone, a conduit through which spiritual forces flow.

**Conclusions:**

Traditional cultural healing remains important to psychiatry because it defies explanation in our usual paradigm. Spiritual transformation and radical empathy may be necessary, though not sufficient components. For the person who undergoes a profound spiritual transformation, extensive changes in self and world view may occur.

**Disclosure:**

No significant relationships.

